# Supplementary material for: Systems-level analyses dissociate genetic regulators of reactive oxygen species and energy production
Source: Proc Natl Acad Sci U S A. 2024 Jan 11;121(3):e2307904121. doi: 10.1073/pnas.2307904121 (PMC10801874; doi:10.1073/pnas.2307904121)
Supplement: Supplementary file 1 — Appendix 01 (PDF) [file pnas.2307904121.sapp.pdf]

Supporting Information for

Systems-level analyses dissociate genetic regulators of reactive oxygen species and energy production

Neal K. Bennett, Megan Lee, Adam L. Orr, Ken Nakamura

Ken Nakamura
Email: Ken.Nakamura@gladstone.ucsf.edu

This PDF file includes:

Figures S1 to S2
Table S1

Other supporting materials for this manuscript include the following:

Datasets S1 to S2

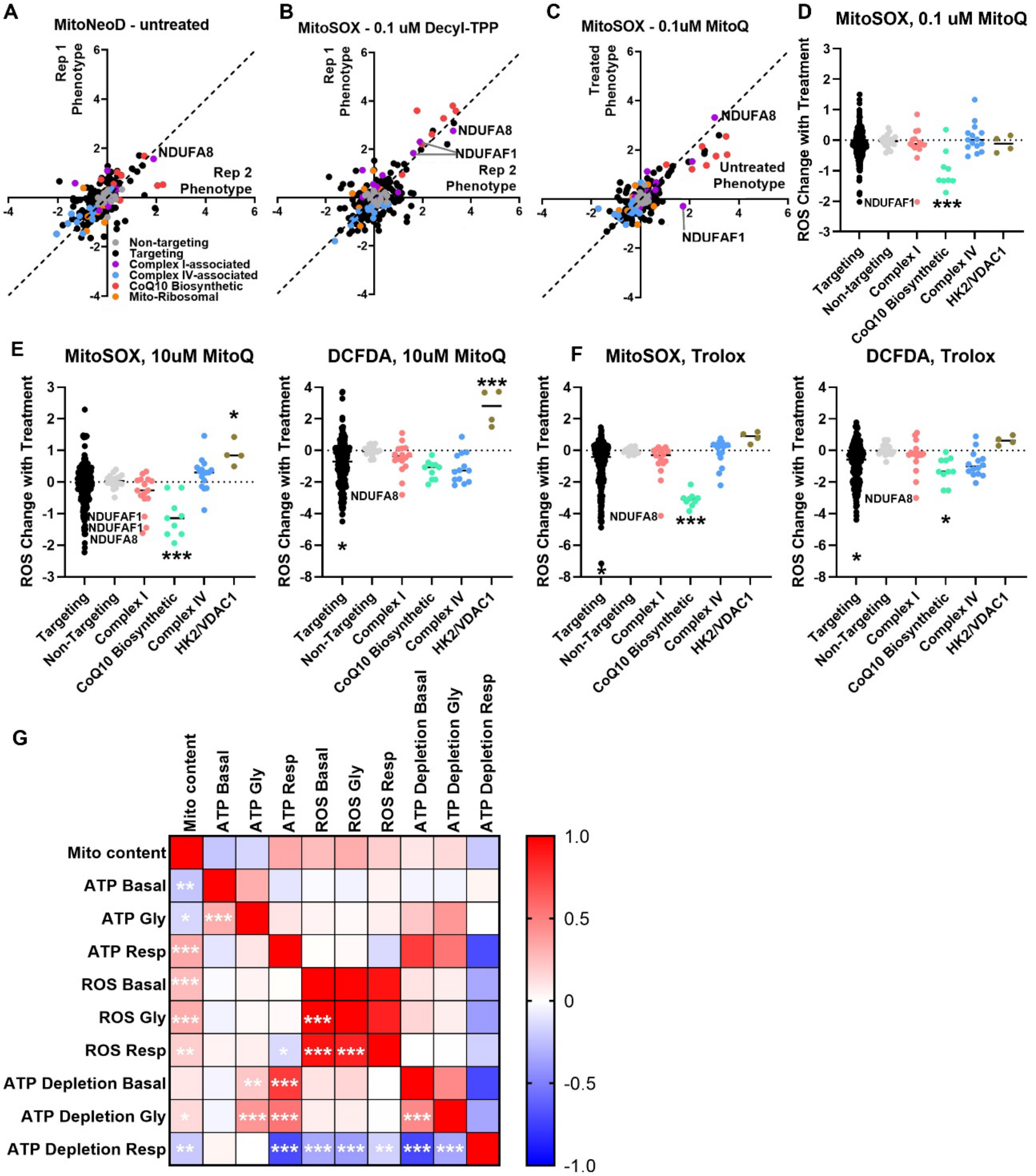

Fig. S1. Antioxidant effect on ROS levels depends on deficient pathways. A) ROS phenotypes of K562 cells expressing a mini-library of CRISPRi sgRNA detected with MitoNeoD were similar to those measured with MitoSOX, with some of the same hit genes like NDUFA8, and similar patterns with complex IV-associated gene knockdowns having low ROS. B) Some of these same patterns were observed in cells treated with 0.1 μM Decyl-TPP, the control for MitoQ Treatment. Cells treated with either C,D) low dose of MitoQ (0.1 μM) or E) high dose (10 μM) of MitoQ respond differently depending on the genes knocked down. MitoQ at both doses abrogated the increase in ROS from CRISPRi knockdown of CoQ10 biosynthetic genes. In contrast, 10 μM MitoQ treatment increased mitochondrial and cytosolic ROS following knockdown of HK2 and VDAC1. F) Trolox (1 mM) abrogated the increase in ROS following knockdown of CoQ10 biosynthetic genes. Trolox treatment also decreased ROS levels across all targeting CRISPRi sgRNA in aggregate. n = 2 replicates. G) Pearson r correlation matrix of mitochondrial content, ATP level and ATP depletion phenotypes in basal, respiration-only, and glycolysis-only conditions collected previously from n =

2 replicates(1), along with ROS phenotypes in the same conditions in cells expressing CRISPRi knockdown libraries. Across ATP level phenotypes, there was only significant correlation between basal and glycolysis-only conditions. In contrast, ROS levels correlated between all substrate conditions.
*$p < 0.05$, **$p < 0.01$, ***$p < 0.001$ by one-way ANOVA with Dunnett's multiple comparisons test (A,B) and (C) by Pearson correlation test.
*$p < 0.05$, **$p < 0.01$, ***$p < 0.001$ by one-way ANOVA with Dunnett's multiple comparisons test (A,B) and (C) by Pearson correlation test.

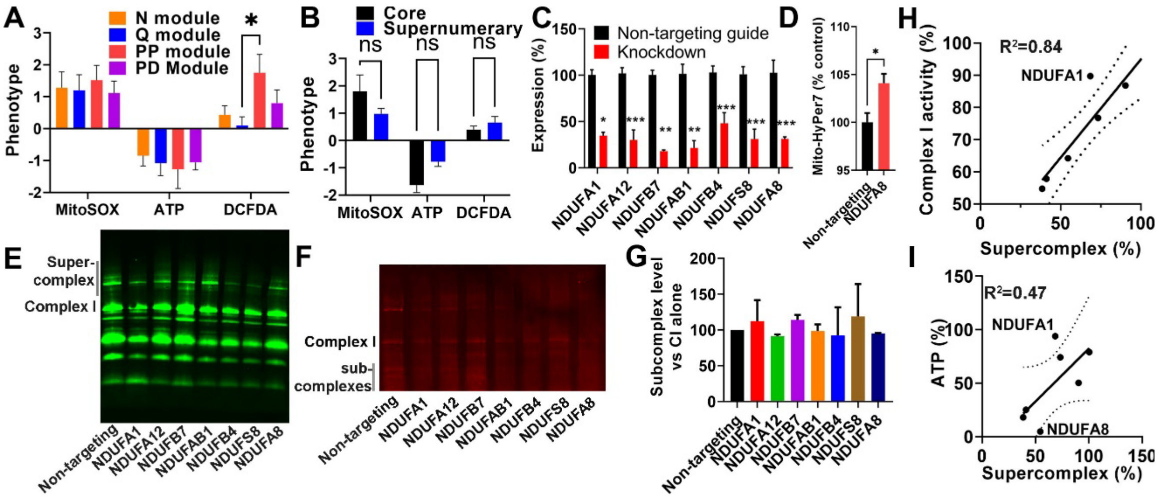

Fig. S2. CRISPRi knockdown of complex I subunits decreases supercomplex levels. A) Knockdown of subunits within functional modules of complex I did not significantly affect either mitochondrial ROS or ATP. Knockdown of subunits within the Q module and PP module significantly differed in their effects on cytosolic ROS. B) Effect of knockdown of core conserved subunits did not significantly differ from the effect of knockdown of non-core or supernumerary subunits on mitochondrial ROS, cytosolic ROS, or ATP. Data compiled from n = 2 experiments. ATP phenotype data previously collected in Bennett et al (1). C) RT-qPCR of K562 lines expressing CRISPRi sgRNA knocking down individual complex I subunits. n = 2-10 replicates per cell line. D) Knockdown of NDUFA8 in K562 cells increases mitochondrial ROS, as measured by mitochondrial matrix-targeted HyPer7. Data compiled from n=2 experiments. E) Additional representative blue-native PAGE gel replicate, loaded with isolated mitochondria for each of seven cell lines expressing CRISPRi knockdown of complex I subunits and a non-targeting control, and stained with total OXPHOS human western blot antibody cocktail. F) Representative blue-native PAGE gel, loaded with isolated mitochondria for each of seven cell lines expressing CRISPRi knockdown of complex I subunits and a non-targeting control, and stained with NDUFS4-targeting antibody to identify NDUFS4-containing sub-complexes at molecular weights less than fully assembled complex I. G) There were no significant differences in subcomplex levels versus non-targeting controls. n = 2 blue-native PAGE gels Supercomplex levels correlate with complex I activity (H) and ATP levels (I). NDUFA1 knockdown has proportionally higher complex I activity and ATP than other subunits, given its level of supercomplex, indicating that its knockdown may preserve a supercomplex species of higher energetic productivity. Trendline shows linear regression across the complex I knockdown cell lines, and the dotted lines are 95% confidence intervals. ATP phenotype data previously collected in Bennett et al (1).
ns = not significant, *p < 0.05, **p < 0.01, ***p < 0.001 by 2-way ANOVA with Tukey's (A,B), or Bonferroni's (C) multiple comparisons test, Student's t-test (D), and one-way ANOVA with Dunnett's multiple comparisons test (G).

Table S1. ROS phenotypes are robust and sensitive to antioxidants.

		Targeting	Non-Targeting
MitoSOX rep1 vs rep2 (Fig 1B, top)	P-value (slope is non-zero)	<0.0001	0.8329
	P-value (slope is not 1)	0.0935	0.0003
	slope	0.9275	0.04461
	slope 95% CI	0.8426 to 1.012	-0.3947 to 0.4839
	R^2	0.6068	0.002693
DCFDA rep1 vs rep2 (Fig 1B, bottom)	P-value (slope is non-zero)	<0.0001	0.7811
	P-value (slope is not 1)	<0.0001	<0.0001
	slope	0.676	0.03889
	slope 95% CI	0.5922 to 0.7599	-0.2518 to 0.3295
	R^2	0.4658	0.004665
MitoSOX 10uM MitoQ (Fig 1C, top left)	P-value (slope is non-zero)	<0.0001	0.3365
	P-value (slope is not 1)	<0.0001	<0.0001
	slope	0.5093	-0.1451
	slope 95% CI	0.4594 to 0.5592	-0.4546 to 0.1644
	R^2	0.5741	0.05441
MitoSOX 1mM Trolox (Fig 1C, top right)	P-value (slope is non-zero)	0.9172	0.5338
	P-value (slope is not 1)	<0.0001	<0.0001
	slope	0.006179	0.08061
	slope 95% CI	-0.1106 to 0.1230	-0.1872 to 0.3484
	R^2	0.00003635	0.02317
DCFDA 10uM MitoQ (Fig 1C, bottom left)	P-value (slope is non-zero)	<0.0001	0.6518
	P-value (slope is not 1)	<0.0001	0.0001
	slope	-0.2437	0.08496
	slope 95% CI	-0.3134 to -0.1740	-0.3053 to 0.4752
	R^2	0.1452	0.01226
DCFDA 1mM Trolox (Fig 1C, bottom right)	P-value (slope is non-zero)	0.2133	0.99
	P-value (slope is not 1)	<0.0001	0.0004
	slope	-0.04547	0.002869
	slope 95% CI	-0.1172 to 0.02628	-0.4739 to 0.4797
	R^2	0.005429	0.00000948
MitoNeoD rep1 vs rep2 (Figure S1A)	P-value (slope is non-zero)	<0.0001	0.173
	P-value (slope is not 1)	<0.0001	0.0112
	slope	0.571	0.3333
	slope 95% CI	0.4800 to 0.6619	-0.1611 to 0.8278
	R^2	0.3489	0.1064
MitoSOX 100 nM Decyl-TPP (Figure S1B)	P-value (slope is non-zero)	<0.0001	0.5415
	P-value (slope is not 1)	<0.0001	0.5415
	slope	0.7423	-0.1019

	slope 95% CI	0.6545 to 0.8300	-0.4469 to 0.2431
	R^2	0.4828	0.02233
MitoSOX 100 nM MitoQ (Figure S1C)	P-value (slope is non-zero)	<0.0001	0.03008
	P-value (slope is not 1)	<0.0001	0.0428
	slope	0.6531	0.3277
	slope 95% CI	0.5923 to 0.7139	-0.09534 to 0.09316
	R^2	0.6194	0.0628

The first column indicates which plot in Figure 1 corresponds to the listed statistics. For both ROS dyes, we find that the slopes are significantly non-zero, indicating significant correlation and robustness, by extra sum-of-squares F-test between phenotypes, n=2 replicates. We also find that for all antioxidant treatments on all ROS dyes, there is a significant effect of the antioxidants on lowering ROS phenotypes for targeting guides by extra sum-of-squares F-test between vehicle and antioxidant treatment, n=2 replicates. Notably, the MitoQ versus vehicle treatment for targeting guides have slopes significantly different than 0, while Trolox versus vehicle are not, indicating that Trolox had a stronger antioxidant effect across all targeting guides.

Dataset S1. Impact of CRISPRi knockdown and antioxidants on ROS phenotypes. K562 cells expressing a mini-library of CRISPRi guides were treated with ROS dyes (MitoSOX , DCFDA, or MitoNeoD) and then sorted by FACS into high- and low- ROS fractions. ROS phenotypes were quantified by measuring guide enrichment in the high versus low ROS fractions (log2 fold-representation scale). Blank cells correspond to guides that did not pass minimum representation thresholds in either cell fraction for a given experimental replicate. N=2 experimental replicates.

Dataset S2. ROS phenotypes following genome-scale CRISPRi knockdowns. Mitochondrial and cytosolic ROS were quantified by measuring guide enrichment (log2 fold representation) in the high versus low ROS cell fractions. Mann-Whitney P-values correspond to the degree of agreement between top guides targeting a given gene. Z-scores were calculated relative to the distribution of pseudogenes, derived from non-targeting control guides included in the library of CRISPRi guides. Hits were determined for genes with either z-scores greater than 3 or less than -3, or with Mann-Whitney P-values less than 0.05. N=2 experimental replicates.

SI References

1. N. K. Bennett *et al.*, Defining the ATPome reveals cross-optimization of metabolic pathways. *Nat Commun* **11**, 4319 (2020).
